# Cooperative *sp* C—H⋯N and *sp*^2^ C—H⋯N hydrogen bonding in the 1:1 cocrystal 1,2-bis­(pyrimidin-5-yl)ethyne–2,6-diethynyl­pyridine

**DOI:** 10.1107/S2056989026011102

**Published:** 2026-10-30

**Authors:** Eric Bosch

**Affiliations:** a Department of Chemistry and Biochemistry Missouri State University, 901 South National Avenue Springfield MO 65897 USA; Texas A & M University, USA

**Keywords:** crystal structure, *sp^2^*-C—H⋯N hydrogen bond, *sp*-C—H⋯N hydrogen bond, *sp^2^*-C—H⋯C(alkyne) inter­action, 2,6-diethynyl pyridine, 1,2-*bis*(pyrimidin-5-yl)ethyne

## Abstract

The title cocrystal C_10_H_6_N_4_·C_9_H_5_N, crystalizes in the monoclinic space group *Pna*2_1_ with two mol­ecules of each component in the asymmetric unit. The extended structure features four unique *sp*-C—H⋯N inter­actions between components that form zigzag hydrogen-bonded chains along the *a-*axis direction. Complementary pyridine and pyrimidine C—H⋯N bonds along the *b-*axis direction result in the formation of planar two-dimensional sheets. A Hirshfeld surface analysis and calculation of inter­mol­ecular inter­action energies within the crystal structure are reported.

## Abstract 2

The title cocrystal crystallizes in the monoclinic space group *Pna*2_1_ with two mol­ecules of each component in the asymmetric unit. The structure contains π-stacked planar two-dimensional sheets with cooperative *sp-*C—H⋯N and *sp^2^*-C—H⋯N hydrogen bonds.

## 1. Chemical context

Early reports that suggested the existence of C—H⋯O hydrogen bonds within crystal structures (Sutor, 1962 ▸) were confirmed and comprehensively reviewed several decades ago (Desiraju, 1996 ▸; Steiner, 2003 ▸). Currently, C—H hydrogen bonding is widely accepted in both chemical and biochemical fields (Derewenda, 2023 ▸). We initially studied terminal alkynes as C—H hydrogen-bond donors since hydrogen atoms bonded to *sp*-C atoms are more polarized, exhibiting a p*K*_a_ of approximately 25. We first focussed on *sp*-C—H⋯N(pyridine) inter­actions and noted that the vast majority of the published examples were reported in crystal structures of single mol­ecules that included both a terminal alkyne and an amine donor. We chose to investigate the potential of these C—H⋯N hydrogen bonds to induce the formation of cocrystals between 1,4-diethynyl benzenes and aromatic amines like 1,2-bis­(4-pyrid­yl)ethyne (Bosch, 2010 ▸, 2026 ▸). We, and others, investigated the role of phenyl C—H⋯N hydrogen bonds in cocrystals between fluorinated benzenes and pyridines (Bosch *et al.*, 2015 ▸; Präsang *et al.*, 2013 ▸). Here we present the cocrystallization of 1,2-bis­(pyrimidin-5-yl)ethyne (**BPE**) with 2,6-diethynyl­pyridine (**DEP**). We expected that we would form cocrystals with π-stacked planar sheets comprising multiple cooperative *sp* and *sp^2^*-C—H⋯N hydrogen bonds. This was based on our knowledge of both *sp-*C—H⋯N hydrogen bonding and *sp^2^-*C—H⋯N hydrogen bonding of pyridines and pyrimidines within the crystal structure of 1,2-bis­(pyrimidin-5-yl)ethyne and its cocrystals with 2,6-*bis*(iodo­ethyn­yl)pyridine (Lang *et al.*, 2024 ▸).
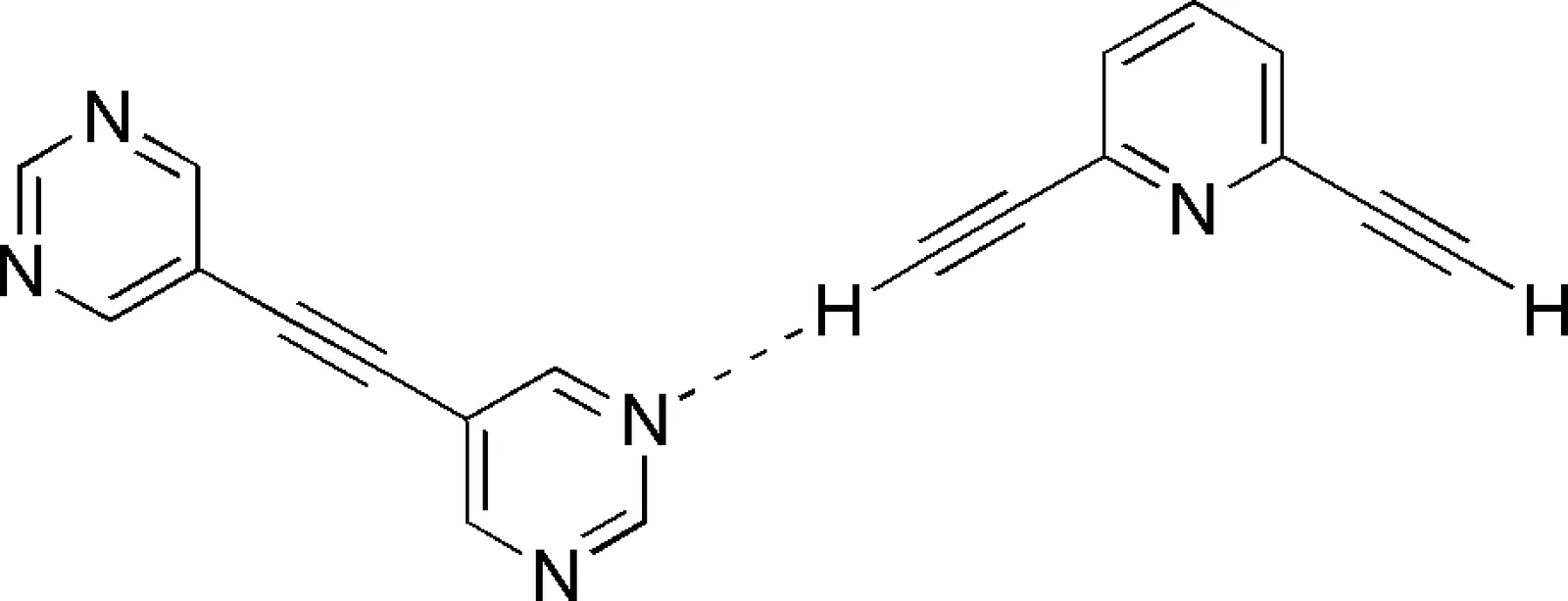


## 2. Structural commentary

**Figure 1:**
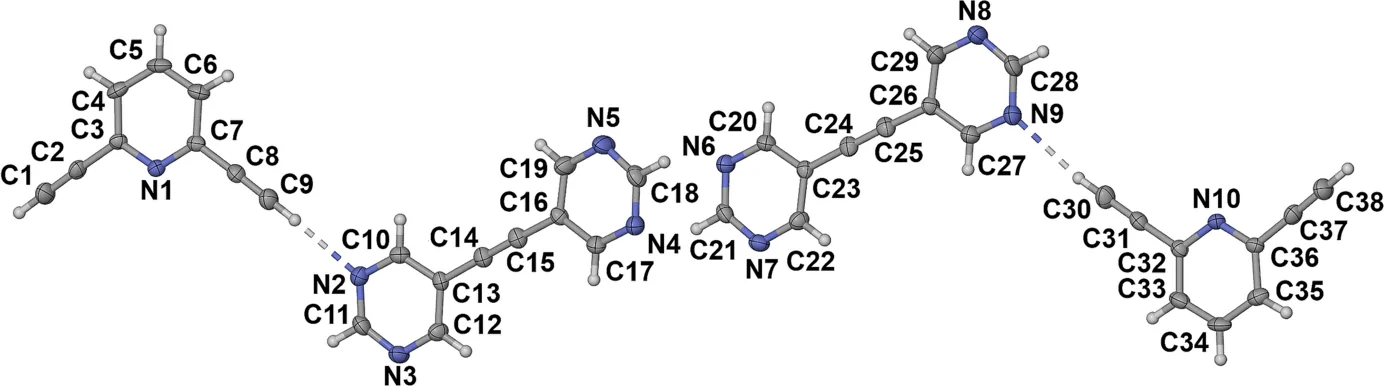
Labeled asymmetric unit of the cocrystal, **BPE·DEP**, with displacement ellipsoids drawn at the 50% level. C—H⋯N hydrogen bonds shown as dashed lines.

The asymmetric unit of the cocrystal **BPE·DEP** contains two unique mol­ecules of each component as shown in Fig. 1 ▸. The two hydrogen-bonded pairs of mol­ecules are in adjacent planes separated by 3.3 Å. The two **BPE** mol­ecules are almost planar with dihedral angles between the pyrimidine rings in each of about 7°. The associated **DEP** mol­ecules are slightly out of the plane defined by each C—H⋯N hydrogen-bonded **BPE**.

## 3. Supra­molecular features

**Figure 2:**
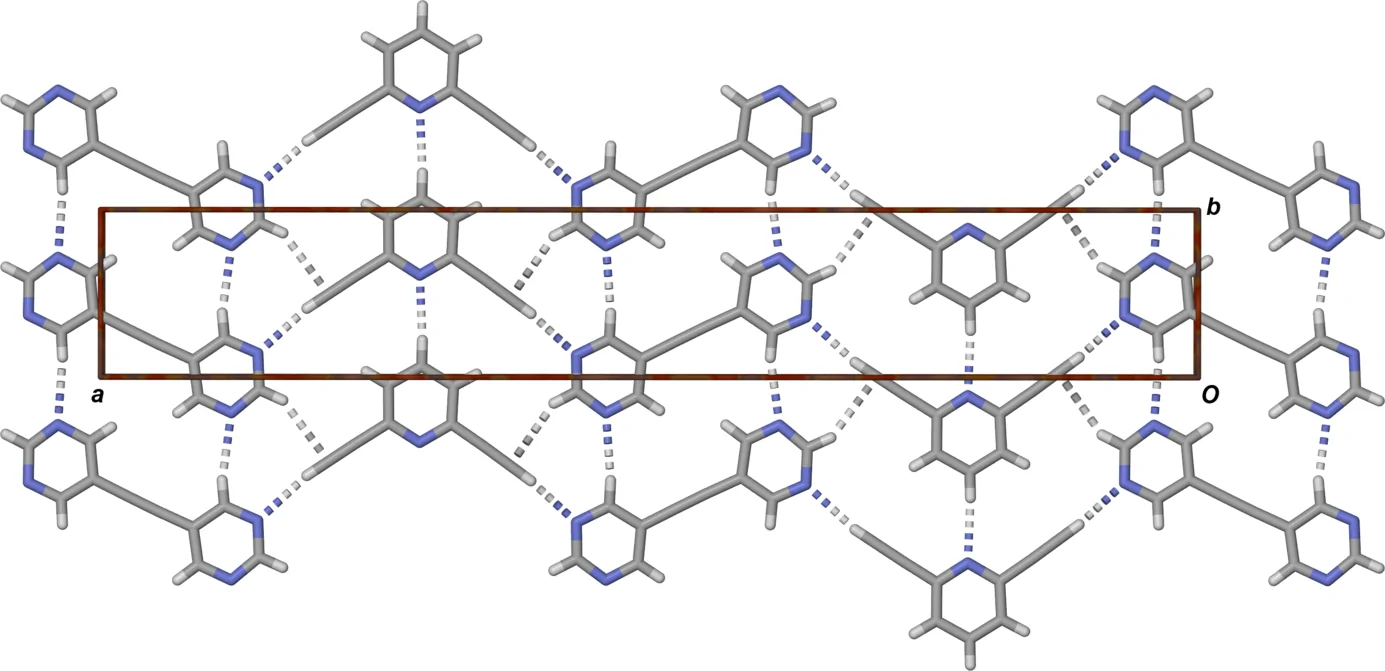
View, along the *c*-axis, of one of the two unique planar sheets of alternating C—H⋯N and C—H⋯C hydrogen bonded mol­ecules within the cocrystal **BPE·DEP**.

The two-dimensional planar sheet of **BPE·DPE** as shown in Fig. 2 ▸ features alternating rows of **BPE** mol­ecules and **DPE** mol­ecules along the direction of the *b* axis. The **BPE** mol­ecules within each row are held together by self-complementary pyrimidine–pyrimidine *sp^2^*-C—H⋯N hydrogen bonds with four hydrogen bonds between each pair of **BPE** mol­ecules. The **DEP** mol­ecules within each row feature a single pyridine-pyridine *sp^2^-*C—H⋯N hydrogen bond. The *sp*-C—H⋯N (pyrimidine) hydrogen bonds effectively zipper the alternating rows together. The ten unique C—H⋯N hydrogen bonds are collated in Table 1 ▸. The first four entries in Table 1 ▸ correspond to the *sp*-C—H⋯N separations that range from 2.46 to 2.51 Å, 84 to 91% of the sum of the van der Waals radii (Bondi, 1964 ▸). There is a weak supporting C—H⋯π inter­action between the pyrimidine C—H between the two N atoms and each alkyne (Fig. 2 ▸) with H⋯C separations ranging from 2.78 to 2.88 Å at 96–99% of the sum of the van der Waals radii. The *sp*-C—H⋯N hydrogen bonds, entries 5 to 8 in Table 1 ▸, have H⋯N separations between 2.32 and 2.47 Å and the pyridine–pyridine *sp^2^*C—H⋯N hydrogen bonds have H⋯N separations of 2.38 and 2.39 Å. These planar sheets are, indeed, like those we observed in the cocrystal between 2,6-*bis*(iodo­ethyn­yl)pyridine and **BPE** (Lang *et al.*, 2024 ▸).

**Figure 3:**
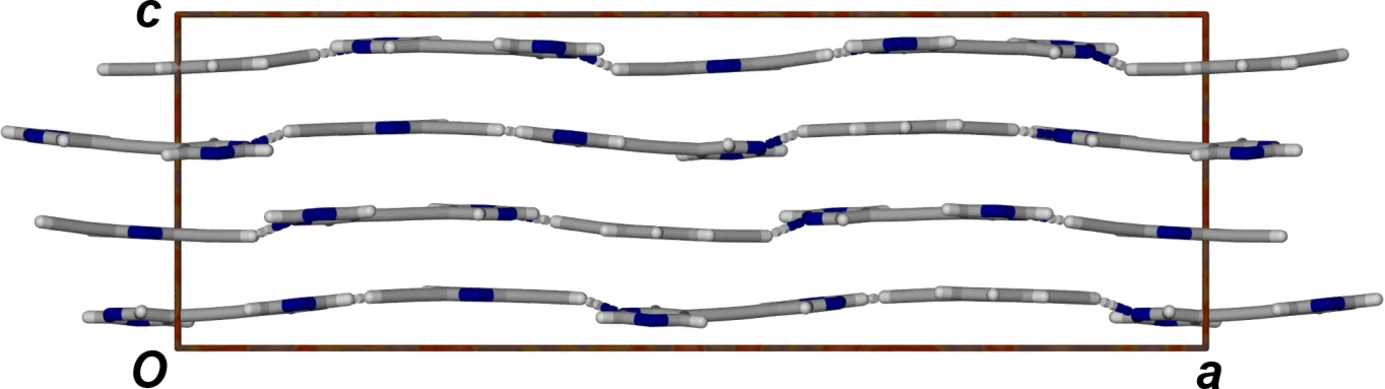
View of the unit cell packing of the cocrystal **BPE·DEP** along the *b* axis.

These planar sheets are π-stacked along the *c*-axis direction as illustrated in Fig. 3 ▸, which shows the slightly corrugated nature of the sheets.

**Figure 4:**
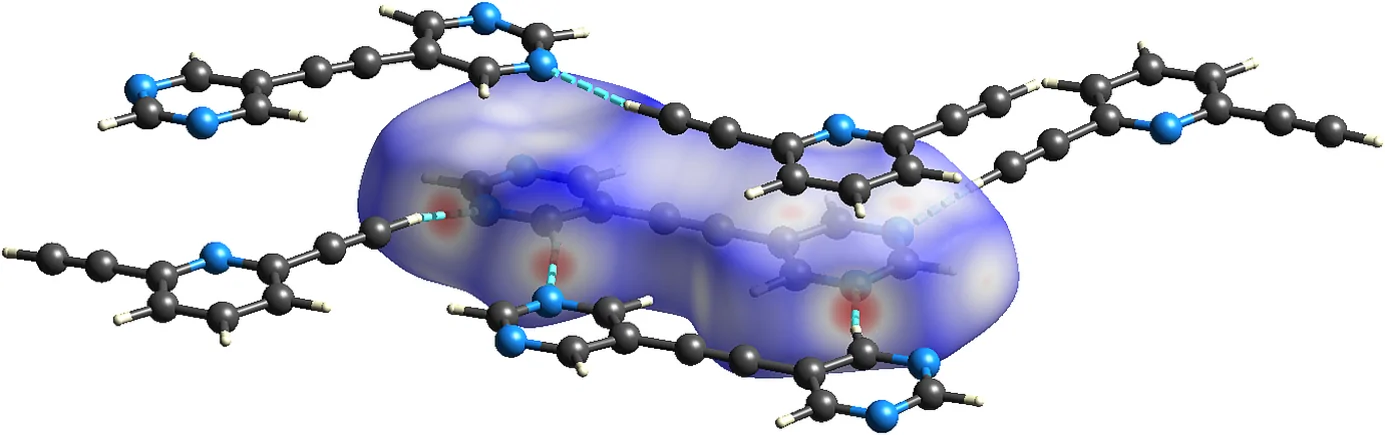
Hirshfeld surface of one of the unique **BPE** mol­ecules and the 5 mol­ecules making close contact. CH⋯N hydrogen bonds shown as dashed lines.

The Hirshfeld surface for one of the unique **BPE** mol­ecules within the cocrystal was calculated with *CrystalExplorer 17* (Spackman *et al.*, 2021 ▸). Fig. 4 ▸ shows the five mol­ecules in close contact to the central **BPE** and highlights the close contacts corresponding to the C—H⋯N hydrogen bonds in red.

**Figure 5:**
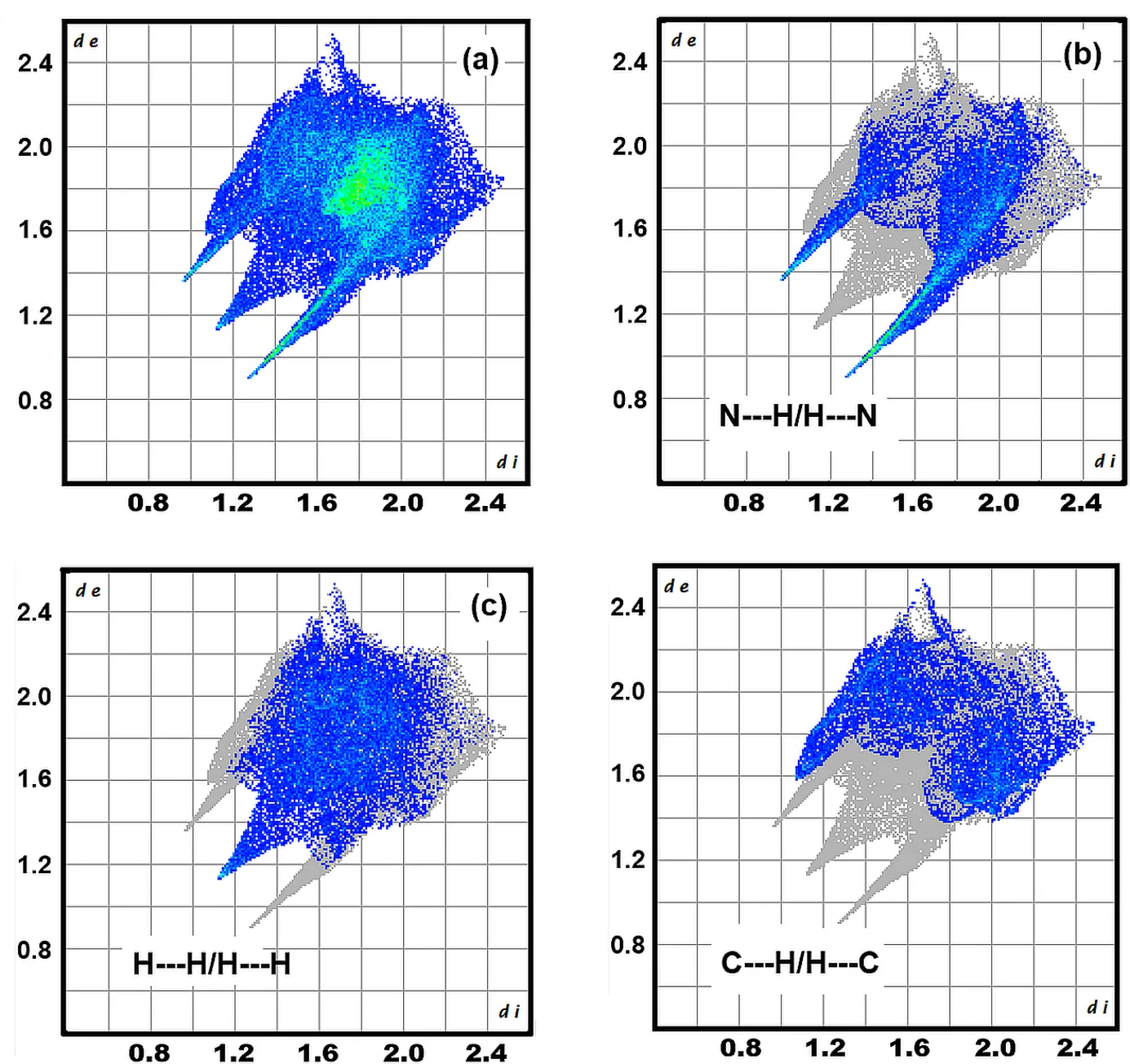
Two-dimensional fingerprint plots showing the contributions of the major inter­actions to the total Hirshfeld surface area of a **BPE** in the cocrystal. (*a*) All atom-atom inter­actions, (*b*) N⋯H/H⋯N inter­actions, (*c*) H⋯H/H⋯H inter­actions and (*d*) C⋯H/H⋯C inter­actions.

The close contacts derived from the Hirshfeld plot were analysed in terms of specific atom-to-atom contacts and the fingerprint plots in Fig. 5 ▸ calculated. The plot corresponding to all atom-to-atom contacts is shown in Fig. 5 ▸(*a*). H⋯H contacts, shown in Fig. 5 ▸(*c*), correspond to 30.5% of the surface area contacts. N⋯H/H⋯N and C⋯H/H⋯C contacts, Fig. 5 ▸(*b*) and 5(*d*), correspond 27.7 and 22.1% of the surface area, respectively. The C⋯C and C⋯N inter­actions that are predominantly π-stacking inter­actions, correspond to 10.3 and 7.8% of the surface area respectively. These latter inter­actions have atom-to-atom distances concentrated around 3.6 Å and appear as the central teal area in Fig. 5 ▸ (*a*).

**Figure 6:**
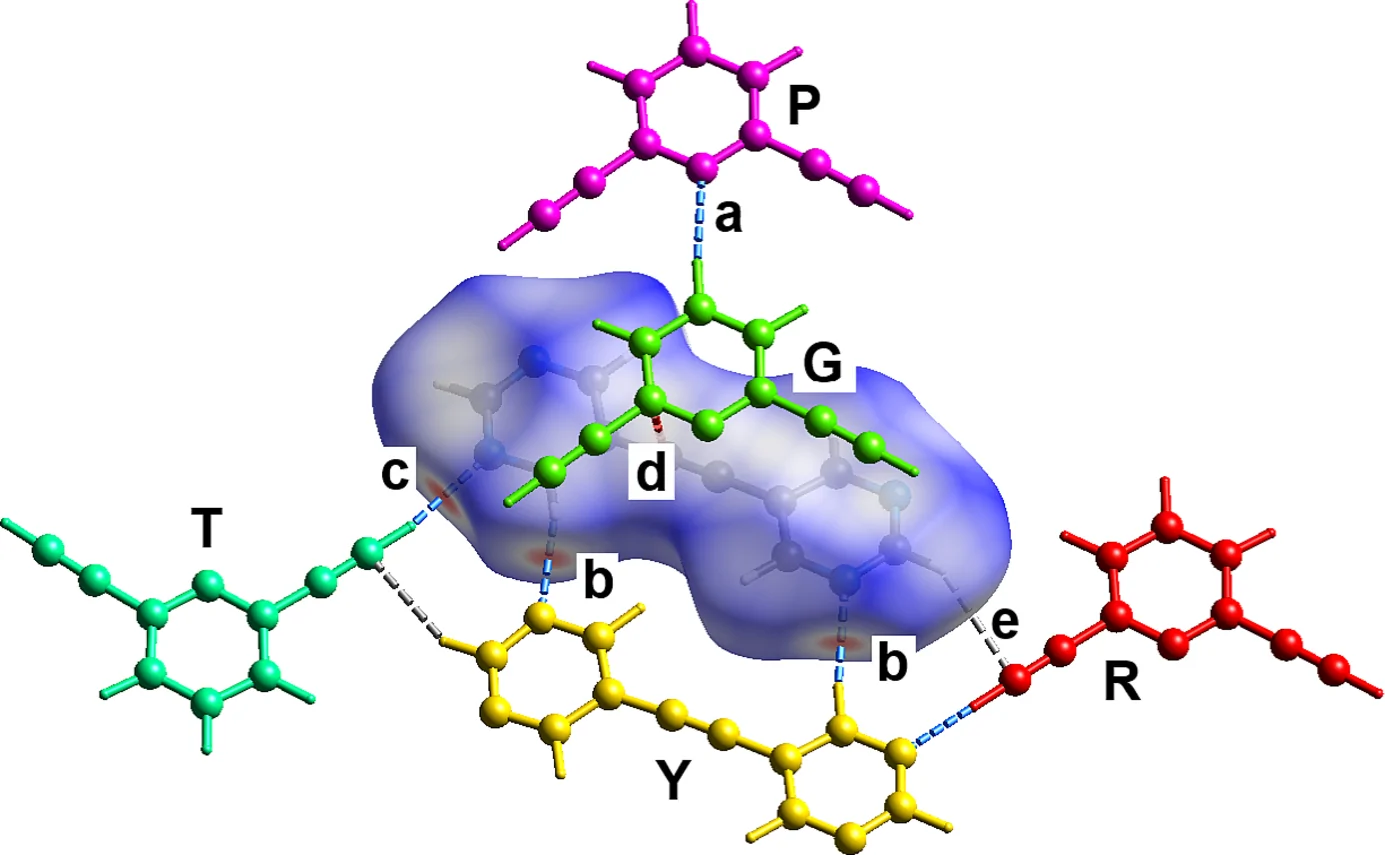
Color-coded mol­ecules, within 3.8 Å of a central **DPE** mol­ecule in cocrystal **(DPE·DEP)**, that have significant attractive inter­molecular energies of inter­action with inter­actions labelled and shown as dashed lines. Labels are; **a** pyridine-pyridine C—H⋯N, **b** pyrimidine-pyrimidine C—H⋯N, **c** alkyne C—H⋯N, **d** π-stacking C⋯C, **e** pyrimidine C—H⋯alkyne. Mol­ecule labels (colours) are: **Y** (yellow), **T** (turquoise), **G** (green), **P** (pink) and **R** (red).

The inter­molecular energy of inter­action of neighbouring mol­ecules within the crystal structure were calculated for each mol­ecule in the asymmetric unit. Each of the major inter­actions is shown in Fig. 6 ▸. Thus the mol­ecule labelled **Y** has two cooperative *sp*^2^-C—H⋯N hydrogen bonds to the central **BPE**, labelled ‘*b*’ in Fig. 6 ▸. The average energy of inter­action between the two **BPE** mol­ecules is −21.4 kJmol^−1^. The **DEP** mol­ecule labelled **T** in Fig. 6 ▸ has an *sp-*C—H⋯N inter­action, labelled ‘c’, with the central **DPE** mol­ecule. The average energy of this inter­action for the four unique alkynes is −12.7 kJmol^−1^. The π-stacked **DEP** mol­ecule labelled **G** has large surface area of inter­action with one alkyne lying along the **BPE** with the close C⋯C contact labelled ‘d’ with an average inter­molecular energy of inter­action of −28.5 kJmol^−1^. The **DEP** mol­ecule labelled **R** has an *sp*^2^-C—H⋯alkyne inter­action labelled ‘e’ with an average energy of inter­action between these mol­ecules of 8.0 kJmol^−1^. The C⋯C separation for ‘e’ is 3.37 Å. Also shown in Fig. 6 ▸ is the *sp*^2^-C—H⋯N hydrogen bond ‘a’ between pyridines labelled **P** and **G** with an average inter­action energy of 12.7 kJmol^−1^.

## 4. Database survey

A search of the Cambridge Crystallographic Database (Version 2026.1.0; Groom *et al.*, 2016 ▸) using Conquest Build 483767 (Bruno *et al.*, 2002 ▸) for structures containing the **DPE** yielded 11 structures. Two of these are the structure of **DPE** alone, refcode AZEDAN (Meitinger *et al.*, 2021 ▸) and AZEDAN01 (Lang *et al.*, 2024 ▸). The coordination complexes formed between **DPE** and copper(I) iodide and bromide, refcodes LUMJEI and LUMJIM, were reported (Georgiev *et al.*, 2001 ▸) as were hydrogen-bonded complexes formed between **DPE** and C-methyl resorcinarene, refcodes PAQYIQ, PAQYOW and PAQYUC (Georgiev *et al.*, 2004 ▸). Recently we formed a halogen-bonded cocrystal of **DPE** with 1,3-di­iodo­tetra­fluoro­benzene, refcode PUZGUP (Nwach­ukwu *et al.*, 2020 ▸) and three halogen-bonded cocrystals of **DPE** with the isomeric bis­(iodo­ethyn­yl)pyridines, refcodes UGUKOA, UGULAN and UGULIV (Lang *et al.*, 2024 ▸). It is noteworthy that self-complementary pyrimidine–pyrimidine C—H⋯N hydrogen bonding, the strongest intermolecular interaction in the cocrystal **DPE·DEP**, is observed in the structures of **DPE** alone and in the halogen-bonded cocrystals of **DPE**. There are three structures of **DEP** in the database. **DEP** alone is refcode IDUWEJ (Ohkita *et al.*, 2001 ▸), the cocrystal of **DEP** with 5-ethynyl­pyrimidine is refcode RUXNAA (Bosch, 2010 ▸). In these structures there are pyridine–pyridine C—H⋯N hydrogen bonds as observed in cocrystal and the cocrystal **DPE·DEP**. Also the cocrystal of **DEP** with *N*-iodo­succinimide is refcode DEGTAO (Bosch & Bowling, 2022 ▸).

## 5. Synthesis and crystallization

The compounds used in this study were available from previous studies (Lang *et al.*, 2024 ▸). Equimolar amounts, 0.1 mmol, of each component were weighed and placed in a small screw-cap vial and 2 mL of di­chloro­methane were added to effect complete solution of both compounds. The lid was loosely attached to permit slow evaporation of the solvent as a homogeneous mass of colourless plates formed.

## 6. Refinement

Crystal data, data collection and structure refinement details are summarized in Table 2 ▸. H atoms were positioned geometrically (C—H = 0.95 Å) and refined as riding with *U*_iso_(H) = 1.2*U*_eq_(C).

## Supplementary Material

Crystal structure: contains datablock(s) I. DOI: 10.1107/S2056989026011102/jy2078sup1.cif

Structure factors: contains datablock(s) I. DOI: 10.1107/S2056989026011102/jy2078Isup2.hkl

Supporting information file. DOI: 10.1107/S2056989026011102/jy2078Isup3.cdx

Supporting information file. DOI: 10.1107/S2056989026011102/jy2078Isup4.cml

CCDC reference: 2598229

Additional supporting information:  crystallographic information; 3D view; checkCIF report

## Figures and Tables

**Table 1:** Hydrogen-bond geometry (Å, °)

*D*—H⋯*A*	*D*—H	H⋯*A*	*D*⋯*A*	*D*—H⋯*A*
C10—H10⋯N3^i^	0.95	2.50	3.423 (6)	165
C17—H17⋯N5^ii^	0.95	2.49	3.420 (6)	166
C20—H20⋯N7^i^	0.95	2.46	3.405 (6)	171
C27—H27⋯N9^ii^	0.95	2.51	3.436 (6)	164
C1—H1⋯N4^iii^	0.95	2.44	3.380 (6)	170
C9—H9⋯N2	0.95	2.33	3.260 (5)	165
C30—H30⋯N8	0.95	2.47	3.380 (6)	160
C38—H38⋯N6^iv^	0.95	2.32	3.268 (6)	175
C5—H5⋯N1^i^	0.95	2.38	3.331 (6)	176
C34—H34⋯N10^ii^	0.95	2.39	3.336 (6)	175

Symmetry codes: (i) 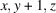; (ii) 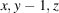; (iii) 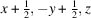; (iv) 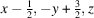.

**Table 2:** Experimental details

Crystal data	
Chemical formula	C_10_H_6_N_4_·C_9_H_5_N
*M*_r_	309.33
Crystal system, space group	Orthorhombic, *Pna*2_1_
Temperature (K)	173
*a*, *b*, *c* (Å)	40.053 (4), 6.1144 (6), 12.9933 (13)
*V* (Å^3^)	3182.1 (5)
*Z*	8
Radiation type	Mo *K*α
μ (mm^−1^)	0.08
Crystal size (mm)	0.60 × 0.20 × 0.05

Data collection	
Diffractometer	Bruker APEXI CCD
Absorption correction	Multi-scan (*SADABS*; Krause *et al.*, 2015 ▸))
*T*_min_, *T*_max_	0.672, 0.746
No. of measured, independent and observed [*I* > 2σ(*I*)] reflections	35545, 6501, 4000
*R*_int_	0.075
(sin θ/λ)_max_ (Å^−1^)	0.625

Refinement	
*R*[*F*^2^ > 2σ(*F*^2^)], *wR*(*F*^2^), *S*	0.049, 0.119, 1.01
No. of reflections	6501
No. of parameters	434
No. of restraints	1
H-atom treatment	H-atom parameters constrained
Δρ_max_, Δρ_min_ (e Å^−3^)	0.20, −0.15

Computer programs: *SMART* and *SAINT* (Bruker, 2014 ▸), *SHELXT2018/2* (Sheldrick, 2015*a* ▸), *SHELXL2019/3* (Sheldrick, 2015*b* ▸) and *X-SEED* (Barbour, 2020 ▸).

## supplementary crystallographic information

### (I) Crystal data

**Table:**

C_10_H_6_N_4_·C_9_H_5_N	*D*_x_ = 1.291 Mg m^−^^3^
*M**_r_* = 309.33	Mo *K*α radiation, λ = 0.71073 Å
Orthorhombic, *P**n**a*2_1_	Cell parameters from 3041 reflections
*a* = 40.053 (4) Å	θ = 2.6–26.0°
*b* = 6.1144 (6) Å	µ = 0.08 mm^−^^1^
*c* = 12.9933 (13) Å	*T* = 173 K
*V* = 3182.1 (5) Å^3^	Plate, colourless
*Z* = 8	0.60 × 0.20 × 0.05 mm
*F*(000) = 1280	

### (I) Data collection

**Table:**

Bruker APEXI CCD diffractometer	6501 independent reflections
Radiation source: fine-focus sealed tube	4000 reflections with *I* > 2σ(*I*)
Graphite monochromator	*R*_int_ = 0.075
Detector resolution: 8.3660 pixels mm^-1^	θ_max_ = 26.4°, θ_min_ = 2.6°
phi and ω scans	*h* = −50→50
Absorption correction: multi-scan (SADABS; Krause *et al.*, 2015))	*k* = −7→7
*T*_min_ = 0.672, *T*_max_ = 0.746	*l* = −16→16
35545 measured reflections	

### (I) Refinement

**Table:**

Refinement on *F*^2^	Hydrogen site location: inferred from neighbouring sites
Least-squares matrix: full	H-atom parameters constrained
*R*[*F*^2^ > 2σ(*F*^2^)] = 0.049	*w* = 1/[σ^2^(*F*_o_^2^) + (0.0462*P*)^2^ + 0.3002*P*] where *P* = (*F*_o_^2^ + 2*F*_c_^2^)/3
*wR*(*F*^2^) = 0.119	(Δ/σ)_max_ < 0.001
*S* = 1.01	Δρ_max_ = 0.20 e Å^−^^3^
6501 reflections	Δρ_min_ = −0.15 e Å^−^^3^
434 parameters	Extinction correction: SHELXL-2019/3 (Sheldrick 2015b)
1 restraint	Extinction coefficient: 0.0008 (2)
Primary atom site location: dual	

### (I) Special details

**Table:**

Geometry. All esds (except the esd in the dihedral angle between two l.s. planes) are estimated using the full covariance matrix. The cell esds are taken into account individually in the estimation of esds in distances, angles and torsion angles; correlations between esds in cell parameters are only used when they are defined by crystal symmetry. An approximate (isotropic) treatment of cell esds is used for estimating esds involving l.s. planes.

### (I) Fractional atomic coordinates and isotropic or equivalent isotropic displacement parameters (Å^2^)

**Table:**

	*x*	*y*	*z*	*U*_iso_*/*U*_eq_	
N1	0.70954 (7)	0.6089 (6)	0.3405 (3)	0.0286 (9)	
C1	0.79271 (11)	0.4959 (7)	0.3440 (4)	0.0453 (12)	
H1	0.812495	0.410429	0.346813	0.054*	
N2	0.56656 (8)	0.1314 (6)	0.3982 (3)	0.0360 (9)	
C2	0.76830 (10)	0.6014 (7)	0.3405 (4)	0.0338 (10)	
N3	0.53964 (9)	−0.2145 (6)	0.4163 (3)	0.0377 (10)	
C3	0.73771 (9)	0.7267 (6)	0.3384 (3)	0.0273 (9)	
N4	0.35714 (8)	0.3586 (6)	0.3636 (3)	0.0387 (9)	
C4	0.73803 (11)	0.9546 (7)	0.3363 (3)	0.0352 (11)	
H4	0.758488	1.032857	0.333021	0.042*	
N5	0.38130 (9)	0.7146 (6)	0.3674 (3)	0.0469 (11)	
C5	0.70769 (11)	1.0643 (8)	0.3393 (4)	0.0404 (11)	
H5	0.707099	1.219661	0.339138	0.048*	
N10	−0.03096 (8)	0.3847 (6)	0.6537 (3)	0.0279 (9)	
C6	0.67862 (11)	0.9464 (7)	0.3425 (4)	0.0369 (11)	
H6	0.657628	1.018548	0.344935	0.044*	
N6	0.32429 (8)	0.6352 (6)	0.5975 (3)	0.0346 (9)	
C7	0.68039 (9)	0.7191 (7)	0.3421 (3)	0.0285 (9)	
N7	0.29795 (9)	0.2857 (6)	0.5884 (3)	0.0354 (9)	
C8	0.65084 (10)	0.5856 (7)	0.3449 (4)	0.0324 (10)	
N8	0.11432 (8)	0.8539 (6)	0.6121 (3)	0.0368 (9)	
C9	0.62710 (10)	0.4715 (7)	0.3483 (4)	0.0400 (11)	
H9	0.607994	0.379638	0.350950	0.048*	
N9	0.13825 (8)	1.2079 (6)	0.5975 (4)	0.0467 (10)	
C10	0.53651 (10)	0.2276 (8)	0.3964 (4)	0.0326 (11)	
H10	0.535352	0.382122	0.389686	0.039*	
C11	0.56605 (11)	−0.0857 (8)	0.4072 (4)	0.0376 (12)	
H11	0.587164	−0.156537	0.407103	0.045*	
C12	0.51034 (11)	−0.1138 (7)	0.4144 (3)	0.0354 (11)	
H12	0.490637	−0.199404	0.420407	0.042*	
C13	0.50719 (10)	0.1103 (7)	0.4040 (3)	0.0288 (10)	
C14	0.47504 (10)	0.2168 (9)	0.3989 (4)	0.0338 (11)	
C15	0.44856 (10)	0.3023 (7)	0.3928 (3)	0.0336 (11)	
C16	0.41605 (10)	0.3996 (7)	0.3822 (3)	0.0292 (10)	
C17	0.38756 (10)	0.2721 (7)	0.3751 (3)	0.0344 (11)	
H17	0.389718	0.117567	0.378542	0.041*	
C18	0.35587 (11)	0.5760 (8)	0.3607 (4)	0.0413 (12)	
H18	0.334343	0.639165	0.352911	0.050*	
C19	0.41140 (11)	0.6223 (8)	0.3783 (4)	0.0435 (13)	
H19	0.430383	0.714761	0.383682	0.052*	
C30	0.05273 (11)	0.4922 (7)	0.6636 (3)	0.0424 (12)	
H30	0.072646	0.576522	0.664071	0.051*	
C31	0.02787 (11)	0.3870 (7)	0.6630 (3)	0.0326 (10)	
C32	−0.00305 (9)	0.2642 (7)	0.6584 (3)	0.0285 (10)	
C33	−0.00334 (12)	0.0375 (7)	0.6581 (4)	0.0365 (11)	
H33	0.016966	−0.042556	0.660930	0.044*	
C34	−0.03355 (11)	−0.0699 (8)	0.6536 (4)	0.0392 (11)	
H34	−0.034419	−0.225118	0.653786	0.047*	
C35	−0.06262 (11)	0.0523 (7)	0.6488 (3)	0.0363 (11)	
H35	−0.083807	−0.017340	0.646112	0.044*	
C36	−0.06015 (10)	0.2790 (7)	0.6481 (3)	0.0290 (10)	
C37	−0.08946 (11)	0.4157 (8)	0.6394 (4)	0.0360 (12)	
C38	−0.11320 (11)	0.5279 (8)	0.6289 (4)	0.0435 (12)	
H38	−0.132307	0.618178	0.620414	0.052*	
C20	0.29428 (9)	0.7296 (7)	0.5965 (4)	0.0318 (11)	
H20	0.292990	0.884667	0.597844	0.038*	
C21	0.32428 (10)	0.4171 (7)	0.5943 (4)	0.0330 (10)	
H21	0.345516	0.347992	0.596533	0.040*	
C22	0.26834 (10)	0.3858 (7)	0.5880 (4)	0.0349 (11)	
H22	0.248720	0.298995	0.583860	0.042*	
C23	0.26504 (9)	0.6106 (7)	0.5935 (3)	0.0269 (9)	
C24	0.23295 (9)	0.7140 (7)	0.5960 (3)	0.0326 (10)	
C25	0.20616 (10)	0.7997 (9)	0.5994 (4)	0.0346 (11)	
C26	0.17343 (10)	0.8939 (7)	0.6015 (4)	0.0302 (10)	
C27	0.14499 (9)	0.7674 (7)	0.6100 (4)	0.0367 (11)	
H27	0.147299	0.613064	0.614459	0.044*	
C28	0.11276 (11)	1.0713 (8)	0.6049 (4)	0.0417 (12)	
H28	0.091100	1.134503	0.605115	0.050*	
C29	0.16841 (11)	1.1186 (8)	0.5953 (4)	0.0419 (12)	
H29	0.187305	1.211648	0.589241	0.050*	

### (I) Atomic displacement parameters (Å^2^)

**Table:**

	*U*^11^	*U*^22^	*U*^33^	*U*^12^	*U*^13^	*U*^23^
N1	0.029 (2)	0.026 (2)	0.0311 (19)	−0.0023 (15)	0.0006 (16)	0.000 (2)
C1	0.029 (2)	0.042 (3)	0.064 (3)	−0.005 (2)	−0.001 (2)	−0.007 (3)
N2	0.030 (2)	0.036 (2)	0.042 (2)	−0.0029 (18)	0.0016 (17)	0.0004 (19)
C2	0.027 (2)	0.033 (3)	0.041 (3)	−0.009 (2)	0.000 (2)	−0.006 (2)
N3	0.033 (2)	0.027 (2)	0.052 (3)	0.0006 (18)	−0.0032 (18)	−0.005 (2)
C3	0.029 (2)	0.023 (2)	0.030 (2)	−0.0036 (18)	−0.0018 (19)	−0.003 (2)
N4	0.029 (2)	0.036 (2)	0.051 (3)	0.0002 (17)	0.0020 (18)	0.002 (2)
C4	0.045 (3)	0.026 (2)	0.035 (3)	−0.012 (2)	0.001 (2)	0.004 (2)
N5	0.036 (2)	0.032 (2)	0.073 (3)	0.0033 (19)	−0.007 (2)	0.003 (2)
C5	0.060 (3)	0.020 (2)	0.041 (3)	0.000 (2)	0.000 (2)	0.001 (3)
N10	0.029 (2)	0.022 (2)	0.033 (2)	−0.0026 (15)	0.0013 (15)	−0.0026 (19)
C6	0.044 (3)	0.024 (2)	0.043 (3)	0.006 (2)	−0.002 (2)	−0.001 (3)
N6	0.029 (2)	0.032 (2)	0.042 (2)	−0.0004 (16)	−0.0013 (18)	0.001 (2)
C7	0.034 (2)	0.024 (2)	0.027 (2)	0.0013 (19)	0.002 (2)	0.000 (2)
N7	0.0378 (19)	0.026 (2)	0.042 (2)	0.0024 (18)	−0.0013 (18)	−0.003 (2)
C8	0.030 (2)	0.031 (3)	0.036 (2)	0.004 (2)	0.000 (2)	0.001 (2)
N8	0.0276 (19)	0.037 (2)	0.046 (2)	0.0010 (17)	0.0017 (17)	−0.002 (2)
C9	0.031 (2)	0.040 (3)	0.049 (3)	0.001 (2)	0.000 (2)	−0.002 (3)
N9	0.033 (2)	0.032 (2)	0.074 (3)	0.0016 (18)	0.004 (2)	−0.004 (2)
C10	0.036 (2)	0.028 (3)	0.034 (3)	0.000 (2)	−0.0024 (18)	0.000 (2)
C11	0.030 (3)	0.036 (3)	0.047 (3)	0.008 (2)	0.003 (2)	−0.003 (2)
C12	0.033 (3)	0.031 (3)	0.042 (3)	−0.005 (2)	−0.004 (2)	−0.001 (2)
C13	0.025 (2)	0.033 (3)	0.028 (2)	0.0022 (19)	0.0020 (19)	−0.003 (2)
C14	0.032 (2)	0.037 (3)	0.033 (3)	0.001 (2)	0.000 (2)	−0.002 (2)
C15	0.032 (2)	0.038 (3)	0.030 (3)	0.002 (2)	−0.0017 (19)	−0.002 (2)
C16	0.028 (2)	0.031 (3)	0.029 (2)	0.0044 (18)	−0.0033 (17)	0.000 (2)
C17	0.035 (2)	0.030 (3)	0.039 (3)	0.002 (2)	0.0013 (19)	0.003 (2)
C18	0.033 (2)	0.043 (3)	0.048 (3)	0.011 (2)	0.000 (2)	0.003 (3)
C19	0.036 (3)	0.034 (3)	0.060 (3)	−0.004 (2)	−0.006 (2)	0.002 (3)
C30	0.031 (2)	0.048 (3)	0.048 (3)	−0.002 (2)	−0.002 (2)	−0.008 (2)
C31	0.031 (2)	0.032 (3)	0.034 (2)	0.008 (2)	0.0011 (19)	−0.001 (2)
C32	0.028 (2)	0.029 (2)	0.028 (2)	0.003 (2)	0.0002 (17)	0.001 (2)
C33	0.042 (3)	0.027 (2)	0.040 (3)	0.008 (2)	0.004 (2)	0.000 (3)
C34	0.057 (3)	0.018 (2)	0.042 (3)	0.001 (2)	0.001 (2)	0.003 (2)
C35	0.043 (3)	0.029 (3)	0.037 (3)	−0.007 (2)	0.000 (2)	0.001 (2)
C36	0.032 (2)	0.023 (2)	0.032 (3)	0.001 (2)	0.0018 (19)	−0.003 (2)
C37	0.028 (2)	0.036 (3)	0.044 (3)	−0.008 (2)	−0.001 (2)	−0.003 (2)
C38	0.029 (2)	0.041 (3)	0.060 (3)	0.002 (2)	−0.003 (2)	0.000 (3)
C20	0.033 (2)	0.025 (3)	0.036 (3)	0.002 (2)	0.001 (2)	0.001 (2)
C21	0.029 (2)	0.034 (3)	0.035 (2)	0.0079 (19)	0.003 (2)	0.000 (2)
C22	0.032 (2)	0.033 (3)	0.040 (3)	−0.004 (2)	−0.004 (2)	0.001 (2)
C23	0.025 (2)	0.030 (2)	0.026 (2)	0.0014 (18)	0.0002 (19)	0.003 (2)
C24	0.032 (2)	0.037 (3)	0.029 (2)	0.002 (2)	0.000 (2)	0.005 (2)
C25	0.034 (2)	0.037 (3)	0.032 (3)	0.003 (2)	0.000 (2)	0.004 (2)
C26	0.026 (2)	0.030 (2)	0.034 (2)	0.0014 (18)	−0.004 (2)	−0.002 (2)
C27	0.034 (2)	0.031 (3)	0.046 (3)	0.001 (2)	0.001 (2)	0.000 (3)
C28	0.028 (2)	0.041 (3)	0.056 (3)	0.006 (2)	0.003 (2)	−0.007 (3)
C29	0.032 (2)	0.033 (3)	0.061 (3)	−0.004 (2)	0.003 (2)	−0.005 (3)

### (I) Geometric parameters (Å, º)

**Table:**

N1—C3	1.339 (5)	C12—C13	1.383 (6)
N1—C7	1.348 (5)	C12—H12	0.9500
C1—C2	1.172 (6)	C13—C14	1.445 (6)
C1—H1	0.9500	C14—C15	1.185 (6)
N2—C11	1.333 (5)	C15—C16	1.438 (6)
N2—C10	1.339 (5)	C16—C19	1.376 (6)
C2—C3	1.445 (5)	C16—C17	1.385 (6)
N3—C11	1.324 (5)	C17—H17	0.9500
N3—C12	1.325 (5)	C18—H18	0.9500
C3—C4	1.394 (5)	C19—H19	0.9500
N4—C18	1.331 (5)	C30—C31	1.185 (6)
N4—C17	1.337 (5)	C30—H30	0.9500
C4—C5	1.389 (6)	C31—C32	1.450 (6)
C4—H4	0.9500	C32—C33	1.386 (6)
N5—C18	1.328 (5)	C33—C34	1.378 (6)
N5—C19	1.339 (5)	C33—H33	0.9500
C5—C6	1.370 (6)	C34—C35	1.385 (6)
C5—H5	0.9500	C34—H34	0.9500
N10—C36	1.338 (5)	C35—C36	1.389 (6)
N10—C32	1.340 (5)	C35—H35	0.9500
C6—C7	1.392 (6)	C36—C37	1.446 (6)
C6—H6	0.9500	C37—C38	1.180 (6)
N6—C20	1.334 (5)	C38—H38	0.9500
N6—C21	1.334 (5)	C20—C23	1.379 (5)
C7—C8	1.438 (5)	C20—H20	0.9500
N7—C21	1.328 (5)	C21—H21	0.9500
N7—C22	1.335 (5)	C22—C23	1.383 (6)
C8—C9	1.180 (6)	C22—H22	0.9500
N8—C28	1.334 (5)	C23—C24	1.433 (5)
N8—C27	1.338 (5)	C24—C25	1.195 (5)
C9—H9	0.9500	C25—C26	1.432 (5)
N9—C28	1.322 (5)	C26—C27	1.381 (5)
N9—C29	1.326 (5)	C26—C29	1.391 (6)
C10—C13	1.380 (5)	C27—H27	0.9500
C10—H10	0.9500	C28—H28	0.9500
C11—H11	0.9500	C29—H29	0.9500
			
C3—N1—C7	117.5 (4)	N5—C18—H18	116.3
C2—C1—H1	180.0	N4—C18—H18	116.3
C11—N2—C10	115.2 (4)	N5—C19—C16	122.9 (4)
C1—C2—C3	178.2 (5)	N5—C19—H19	118.5
C11—N3—C12	115.5 (4)	C16—C19—H19	118.5
N1—C3—C4	123.1 (4)	C31—C30—H30	180.0
N1—C3—C2	115.4 (4)	C30—C31—C32	177.4 (5)
C4—C3—C2	121.5 (4)	N10—C32—C33	122.9 (4)
C18—N4—C17	115.7 (4)	N10—C32—C31	115.5 (4)
C5—C4—C3	118.3 (4)	C33—C32—C31	121.7 (4)
C5—C4—H4	120.8	C34—C33—C32	118.9 (4)
C3—C4—H4	120.8	C34—C33—H33	120.5
C18—N5—C19	115.4 (4)	C32—C33—H33	120.5
C6—C5—C4	119.3 (4)	C33—C34—C35	118.9 (4)
C6—C5—H5	120.3	C33—C34—H34	120.5
C4—C5—H5	120.3	C35—C34—H34	120.5
C36—N10—C32	117.7 (4)	C34—C35—C36	118.6 (4)
C5—C6—C7	118.8 (4)	C34—C35—H35	120.7
C5—C6—H6	120.6	C36—C35—H35	120.7
C7—C6—H6	120.6	N10—C36—C35	123.0 (4)
C20—N6—C21	115.6 (4)	N10—C36—C37	115.7 (4)
N1—C7—C6	122.9 (4)	C35—C36—C37	121.3 (4)
N1—C7—C8	115.4 (4)	C38—C37—C36	177.8 (5)
C6—C7—C8	121.7 (4)	C37—C38—H38	180.0
C21—N7—C22	115.4 (4)	N6—C20—C23	122.5 (4)
C9—C8—C7	178.2 (5)	N6—C20—H20	118.7
C28—N8—C27	115.8 (4)	C23—C20—H20	118.7
C8—C9—H9	180.0	N7—C21—N6	127.3 (4)
C28—N9—C29	116.4 (4)	N7—C21—H21	116.3
N2—C10—C13	122.4 (4)	N6—C21—H21	116.3
N2—C10—H10	118.8	N7—C22—C23	122.7 (4)
C13—C10—H10	118.8	N7—C22—H22	118.6
N3—C11—N2	127.7 (4)	C23—C22—H22	118.6
N3—C11—H11	116.1	C20—C23—C22	116.4 (4)
N2—C11—H11	116.1	C20—C23—C24	121.9 (4)
N3—C12—C13	122.9 (4)	C22—C23—C24	121.7 (4)
N3—C12—H12	118.6	C25—C24—C23	179.1 (5)
C13—C12—H12	118.6	C24—C25—C26	177.5 (6)
C10—C13—C12	116.4 (4)	C27—C26—C29	116.0 (4)
C10—C13—C14	121.4 (4)	C27—C26—C25	122.1 (4)
C12—C13—C14	122.2 (4)	C29—C26—C25	121.9 (4)
C15—C14—C13	178.7 (5)	N8—C27—C26	122.5 (4)
C14—C15—C16	177.6 (5)	N8—C27—H27	118.7
C19—C16—C17	116.3 (4)	C26—C27—H27	118.7
C19—C16—C15	122.4 (4)	N9—C28—N8	126.8 (4)
C17—C16—C15	121.3 (4)	N9—C28—H28	116.6
N4—C17—C16	122.4 (4)	N8—C28—H28	116.6
N4—C17—H17	118.8	N9—C29—C26	122.5 (4)
C16—C17—H17	118.8	N9—C29—H29	118.8
N5—C18—N4	127.3 (4)	C26—C29—H29	118.8

### (I) Hydrogen-bond geometry (Å, º)

**Table:**

*D*—H···*A*	*D*—H	H···*A*	*D*···*A*	*D*—H···*A*
C10—H10···N3^i^	0.95	2.50	3.423 (6)	165
C17—H17···N5^ii^	0.95	2.49	3.420 (6)	166
C20—H20···N7^i^	0.95	2.46	3.405 (6)	171
C27—H27···N9^ii^	0.95	2.51	3.436 (6)	164
C1—H1···N4^iii^	0.95	2.44	3.380 (6)	170
C5—H5···N1^i^	0.95	2.38	3.331 (6)	176
C9—H9···N2	0.95	2.33	3.260 (5)	165
C30—H30···N8	0.95	2.47	3.380 (6)	160
C34—H34···N10^ii^	0.95	2.39	3.336 (6)	175
C38—H38···N6^iv^	0.95	2.32	3.268 (6)	175

Symmetry codes: (i) *x*, *y*+1, *z*; (ii) *x*, *y*−1, *z*; (iii) *x*+1/2, −*y*+1/2, *z*; (iv) *x*−1/2, −*y*+3/2, *z*.
